# The Multi-Component Causes of Late Neonatal Sepsis—Can We Regulate Them?

**DOI:** 10.3390/nu14020243

**Published:** 2022-01-07

**Authors:** Magdalena Pilarczyk-Zurek, Grzegorz Majka, Beata Skowron, Agnieszka Baranowska, Monika Piwowar, Magdalena Strus

**Affiliations:** 1 Department of Microbiology, Faculty of Biochemistry, Biophysics and Biotechnology, Jagiellonian University, 30-387 Cracow, Poland; magdalena.pilarczyk-zurek@uj.edu.pl; 2 Chair of Microbiology, Faculty of Medicine, Jagiellonian University Medical College, 31-121 Cracow, Poland; grzegorz.majka@uj.edu.pl; 3 Chair of Immunology, Faculty of Medicine, Jagiellonian University Medical College, 31-121 Cracow, Poland; 4 Medical Department Diagnostyka S.A., 31-864 Cracow, Poland; beata.skowron1983@gmail.com; 5 Chair of Pathophysiology, Faculty of Medicine, Jagiellonian University Medical College, 31-121 Cracow, Poland; agnieszka.1.baranowska@uj.edu.pl; 6 Department of Bioinformatics and Telemedicine, Faculty of Medicine, Jagiellonian University Medical College, 30-688 Cracow, Poland; monika.piwowar@uj.edu.pl

**Keywords:** manganese lactoferrin, neonatal infection, rat sepsis model

## Abstract

Elucidating the mechanisms of bacterial translocation is crucial for the prevention and treatment of neonatal sepsis. In the present study, we aimed to evaluate the potential of lactoferrin to inhibit the development of late-onset blood infection in neonates. Our investigation evaluates the role of key stress factors leading to the translocation of intestinal bacteria into the bloodstream and, consequently, the development of life-threatening sepsis. Three stress factors, namely weaning, intraperitoneal administration of Gram-positive cocci and oral intake of Gram-negative rods, were found to act synergistically. We developed a novel model of rat pups sepsis induced by bacterial translocation and observed the inhibition of this process by supplementation of various forms of lactoferrin: iron-depleted (apolactoferrin), iron-saturated (hololactoferrin) and manganese-saturated lactoferrin. Additionally, lactoferrin saturated with manganese significantly increases the *Lactobacillus* bacterial population, which contributes to the fortification of the intestinal barrier and inhibits the translocation phenomenon. The acquired knowledge can be used to limit the development of sepsis in newborns in hospital neonatal intensive care units.

## 1. Introduction

Neonatal sepsis still represents an important cause of mortality and morbidity among infants [1]. Rapid deaths of newborns primarily result from the extremely short time that may elapse from the onset of an acute infection to the development of bloodstream infection (BSI) and sepsis. According to ECDC data (2011–2012, based on the European Centre for Disease Prevention and Control data), bloodstream infections in newborns are responsible for over 44.6% of all healthcare-associated infections [2]. Neonatal sepsis may be categorized as early onset sepsis (EOS) or late-onset sepsis (LOS). Early onset sepsis appears up to 72 h after delivery and is usually associated with colonization by high-virulence etiological factors, from both the maternal vaginal (*Streptococcus agalactiae* and *Streptococcus pyogenes*) and gastrointestinal (GI) microbiota (*Escherichia coli* and other Gram-negatives rods [3]. In contrast, late-onset sepsis is due to immature immunity including the skin, respiratory and gastrointestinal (GI) systems, need for invasive procedures, environmental exposures and prolonged hospital stay. In this case, positive blood cultures may be associated with both catheter-associated infection, nosocomial transmission and colonization factors [4].

Our attention has been focused on bloodstream infections originating from the gastrointestinal tract in very low birth weight infants. Bacterial translocation (BT), defined as invasion of indigenous intestinal bacteria through the mucosa into tissues, might therefore contribute to the development of sepsis [5]. Bacterial translocation from the gastrointestinal tract (GI) is an important pathway initiating late neonatal sepsis. The newborn’s own microbiota, depending on its qualitative and quantitative composition, external stress factors, immaturity of intestinal epithelium and mucosal immunity, as well as suboptimal nutrition, in particular lack of breast milk feeding, play major roles in facilitating bacterial translocation [2]. On the other side, there are reports claiming that supplementation with lactoferrin, probiotics or prebiotics could serve to prevent bacterial translocation in neonates and inhibit the development of sepsis [5].

Understanding the pathomechanism and methods of counteracting LOS in newborns is still very difficult due to the lack of an appropriate animal model. There have been several established neonatal rodent models, e.g., with stress induced by weaning, intestinal bacterial translocation and induced sepsis [6]. In rat pups, mother separation is a stress factor that stimulates immune response, increases levels of inflammatory triggers and often leads to gut microbiota distribution [7]. Neonate rats at the 12th postnatal day can be used as a model for late sepsis and preterm human newborns because of morphologically immature intestine with high permeability [8].

The aim of our work was to investigate the factors that the phenomenon of bacterial translocation depends on, as well as the mechanisms regulating intestinal leakage and ways to reduce the risk of late sepsis by feeding newborns. We strived to establish an experimental model of bacterial translocation induced by systemic inflammation triggered by intraperitoneal staphylococcal infection following the oral administration of *Escherichia coli.* Our study has shown that oral administration of various form of lactoferrin in the established model can prevent detrimental bacterial translocation. All tested forms, i.e., iron-depleted, iron-saturated and manganese-saturated lactoferrin, mitigated the translocation of *E. coli* from intestinal lumen to blood. Additionally, manganese-saturated protein had a positive impact on the population numbers on lactobacilli.

## 2. Materials and Methods

### 2.1. Animals

For a better understanding of the pathomechanism and for evaluating methods of counteracting LOS in newborns, we developed a new rat model and used it in the present research. Animal protocols conformed with the guidelines from Directive 2010/63/EU of the European Parliament on the protection of animals used for scientific purposes and approved by the Jagiellonian University Ethical Committee on Animal Experiments (no. 190/2012). Male albino Wistar rat pups [WistarKrf (Wi) Wu)] with a 20–30 g body weight were used in the experiment. Animals were housed in standard cages in temperature-controlled rooms (18–22 °C, 50–60% humidity) under a 12 h light cycle (06:00–18:00) and appropriate environmental enrichment was introduced in the cages. Standard laboratory pelleted formula (Labofeed B, Pasze Kcynia, Poland, containing 25% protein, 8% fat and 67% carbohydrates) and tap water were provided ad libitum. The entire process of developing our model required many preliminary experiments to determine the fastest weaning time, body weight of pups or the choice of a day of the experiment in which the phenomenon of intestinal translocation typical of LOS would take place.

Based on the pilot study results, day 12 was selected as appropriate for weaning and starting the experiment. From that day on, the animals can live independently. The possibility of taking water and food was additionally facilitated for the animals: bottles with an elongated cap were used, and the food was kept in the cage in a crumbled form, adapted to the size of the animal. The reduction in the amount of food and traces of its bites were observed daily. All animals, despite significant weight loss and a drop in body temperature, survived the withdrawal period. The experiment carried out provides not only scientific justification, but also practical possibilities of using its results.

### 2.2. Grouping

The first stage involved the experiment using 48 pups, which were randomly assigned to the experimental groups: non-weaned control group (NW, *n* = 6), weaned group (W, *n* = 6), weaned pups with intraperitoneal administration of *Staphylococcus haemolyticus* 186 (W+Sh, *n* = 6), weaned pups with oral administration of *Escherichia coli* 3A/1 (W+Ec, *n* = 6) and target model weaned group with administration of both intraperitoneal *S. haemolyticus* 186 and oral *E. coli* 3A/1 (W+Sh+Ec, *n* = 24).

The second stage of the study included 42 male pups that served as the model group (W+Sh+Ec), and were used to study the effect of oral administration of various lactoferrin forms for the following groups: metal-depleted apoLf (W+Sh+Ec+apoLf, *n* = 12), iron-saturated holoLf (W+Sh+Ec+holoLf, *n* = 12), manganese-saturated MnLf (W+Sh+Ec+MnLf, *n* = 12).

### 2.3. Procedures Performed on Animals

Weaning. For 11 days after birth, pups remained with the mother and were fed mother’s milk. On day 12 of the experiment, pups were weaned and transferred to separate cages (max. 6 pups per cage).

Temperature and weight measurement. The temperature from anus and weight of the animals were monitored on days 12, 15, 16 and 17 at 9:00 a.m.

*Staphylococcus* administration. Selected strain: coagulase-negative *Staphylococcus haemolyticus* 186 was isolated from blood of a very low birth weight neonate with clinical symptoms of sepsis. Utilization of this strain was approved by the Bioethics Committee of Jagiellonian University Medical College (opinion no. KBET/221/B/2011). Bacterial strain was inoculated into 10 mL liquid TSB broth (Becton Dickinson, Franklin Lakes, NJ, USA). The cultures were carried out overnight at 37 °C. After a double wash (10,000 rpm, 3 min, RT), the obtained pellets were suspended in PBS, the OD was adjusted to correspond to 1 × 10^9^ cfu/mL and a volume of 200 µL (dose of 2 × 10^8^ cfu) was administered intraperitoneally on day 15 to pups in groups W+Sh and W+ Sh+Ec (Table 1.)

*Escherichia coli* administration. Selected strain: *Escherichia coli* 3A/1 was isolated from an infant after approval by the Bioethics Committee of Jagiellonian University Medical College (no. KBET/75/B from 15 November 2007). The strain was selected based on the frequency analyses of the genes responsible for synthesis of proteins for acquisition of iron ions [9,10]. Bacterial cultures were inoculated into 10 mL liquid TSB broth (Becton Dickinson, USA). The culture was carried out overnight at 37 °C. After a double wash (10,000 rpm, 3 min, RT), the obtained pellets were suspended in PBS, the OD adjusted to correspond to 1 × 10^9^ cfu/mL and a volume of 100 µL (dose of 1 × 10^8^ cfu) was administered orally, twice on day 16 to pups in groups W+Ec and W+Ec+Sh (Table 1).

Blood collection. To collect blood for microbial culture, a drop of blood was taken from the incised tip of the tail. This procedure was performed on days 15 and 16, 4 h after administration of the bacterial suspensions to the animals.

Lactoferrin administration. On days 14–16, lactoferrin forms differing in metal saturation were administered twice a day orally at a dose of 300 mg/kg/day (every day between 8 and 9 a.m. and 14 and 15 p.m. in the treatment room) in appropriate groups. The modified lactoferrin forms were used: iron-depleted (apolactoferrin, apoLf, 1.2 ± 0.2% Fe saturation), iron-saturated (hololactoferrin, holoLf, 71.8 ± 6.5% Fe saturation) and manganese-saturated lactoferrin (MnLf, 1.2 ± 0.2% Fe saturation, 47.1 ± 2.0% Mn^3+^ saturation) [11].

Intestinal permeability was assessed following oral administration of FITC-Dextran (FD4, Sigma-Aldrich, St. Louis, MO, USA). Pups were given FITC-Dextran orally (50 mg/100 g body weight), 4 h before sacrifice. Whole blood was obtained by cardiac puncture at the time of necropsy. FITC-Dextran measurements were performed in a plate reader at a fluorescence excitation of 480 nm and an emission wavelength of 520 nm [12].

Euthanasia and necropsy. On day 17, all animals were euthanized by anesthesia overdose (Pentobarbital, Morbital, Pulawy, route of administration i.p. 100 mg/kg body weight). During necropsy, blood samples, spleen and feces from large intestine were collected. Changes such as colonic congestion, intestinal bloating and colon fragility were assessed.

### 2.4. Clinical Score (CS) as Symptoms of Sepsis

The symptoms of systemic inflammation were evaluated by inspecting changes in motor activity, lethargy, shivering and piloerection. Each one of these conditions was scored as 0 (no observable symptom), 1 (a noticeable symptom) or 2 (a severe symptom). The total score was calculated by adding the individual scores of each parameter. Ranging from 0 (normal) to 8 (serious condition), these were monitored on days 12, 15, 16 and 17.

### 2.5. Bacterial Load as Translocation Evidence

Spleen and feces were aseptically collected and homogenized in sterile PBS. Homogenized tissues were subjected to serial log fold dilutions in sterile saline. The bacterial load in blood, spleens and feces was determined by plating 10-fold serial dilutions on agar plates: Columbia agar medium (SIGMA) with 5% sheep blood for isolation of *Staphylococcus* spp. and on MacConkey agar (BioMaxima, Lublin, Poland) for isolation of *Escherichia coli.* The plates were incubated for 24 h at 37 °C and the colonies were isolated. *Lactobacillus* bacteria were isolated from feces and cultured on MRS plates (Biomaxima) in anaerobic conditions at 37 °C. Translocation was confirmed by positive culture of any enteric *E. coli* strains (either commensal or 3A/1) in the blood or spleen (shown as total *E. coli*). Differentiation of *E. coli* 3A/1 strain vs. commensal bacteria was performed using antibiotic resistance pattern (diffusion-disk method with the used of AMP10 = 14 mm, CXM30 = 19 mm, AMC30 = 19 mm, MXF5 = 22 mm, CIP30 = 25 mm).

### 2.6. Statistical Analysis

For statistical analyses and graphic presentations of the obtained results, the R program [13] with the datarium, ggpubr and rstatix libraries was used. A comparison of mean values across more variables based on repeated observations was conducted by the repeated measures ANOVA. Comparison of means between groups was based on ANOVA with the Tukey test.

## 3. Results

### 3.1. Physiological Effect of the Development of the Disorders in Pups

**Figure 1:**
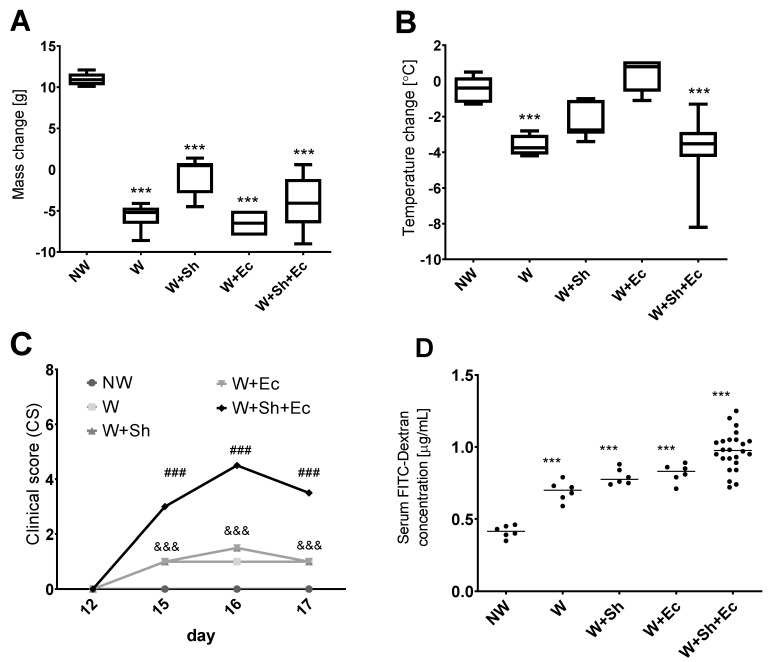
Clinical parameters of animals in tested groups: change in weight (**A**) and temperature (**B**) between the day of necropsy (day 17) and weaning (day 12). (**C**) Clinical scores (reduced motor activity, lethargy, shivering, piloerection) were assessed on days 12, 15, 16 and 17. FITC-Dextran concentration was calculated using standard curve. (**D**) *** *p* < 0.001 vs. NW, ### *p* < 0.001 vs. NW, W, W+Ec and W+Sh, &&& *p* < 0.001 vs. NW and W+Sh+Ec. NW-non-weaned control group, W-weaned group, W+Sh-weaned pups with intraperitoneal administration of *Staphylococcus haemolyticus* 186, W+Ec-weaned pups with oral administration of *Escherichia coli* 3A/1, W+Sh+Ec-target model group weaned with administration of both intraperitoneal *S. haemolyticus* 186 and oral *E. coli* 3A/1.

Body mass differences between day 17 (necropsy) and day 12 (weaning) were analyzed between the experimental groups. All weaned groups (W, W+Sh, W+Ec, W+Sh+Ec) demonstrated significant decrease in the body weight between days 12 and 17, as compared to the non-weaned control group (NW) (Figure 1A). Temperature changes were compared likewise and revealed a significant decrease in temperature between days 12 and 17 for the W and W+Sh+Ec groups in comparison to the non-weaned group (NW) (Figure 1B). Clinical scores on days 15, 16 and 17 were the highest for the W+Sh+Ec group, significantly different from all the other groups. At the same time, the non-weaned group (NW) differed significantly from W, W+Sh and W+Ec groups (Figure 1C).

### 3.2. Bacterial Load as Translocation Evidence

**Figure 2:**
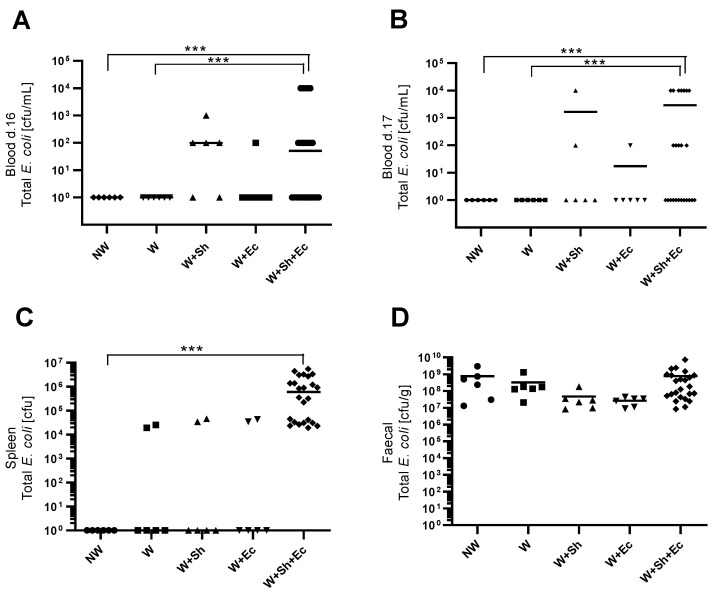
Population numbers of total *E. coli* in blood d. 16 (**A**), blood d. 17 (**B**), spleen (**C**) and feces (**D**). *** *p* < 0.01. NW-non-weaned control group, W-weaned group, W+Sh-weaned pups with intraperitoneal administration of *Staphylococcus haemolyticus* 186, W+Ec-weaned pups with oral administration of *Escherichia coli* 3A/1, W+Sh+Ec-target model group weaned with administration of both intraperitoneal *S. haemolyticus* 186 and oral *E. coli* 3A/1.

Weaning itself did not have a significant impact on the incidence of bacterial translocation from the intestinal lumen to the bloodstream (Figure 2A–C). However, the addition of another stress factor—intraperitoneal or orally delivered bacterial suspension—notably increased population numbers of *E. coli*, both in blood (Figure 2A,B) and spleen (Figure 2C), for respective groups (W+Sh, W+Ec and W+Sh+Ec vs. NW group). This increase was statistically significant only for the group with three stress factors (W+Sh+Ec). All tested animals in the model group (W+Sh+Ec) had *E. coli* present in their spleen compared to none in the NW group and 33% in intermediate groups (W, W+Sh and W+Ec). At the same time, fecal level of total *E. coli* was not altered (Figure 2D). Microbiological data were confirmed by analysis of serum FITC-Dextran concentrations. Unsurprisingly, the non-weaned control group (NW) showed the lowest levels of serum FITC-Dextran (a marker of intestinal translocation), significantly lower than all other groups (Figure 1D). On the other hand, the model group (W+Sh+Ec) had the highest levels of FITC-Dextran in serum, significantly higher than all other groups.

### 3.3. Lactoferrin Supplementation

**Figure 3:**
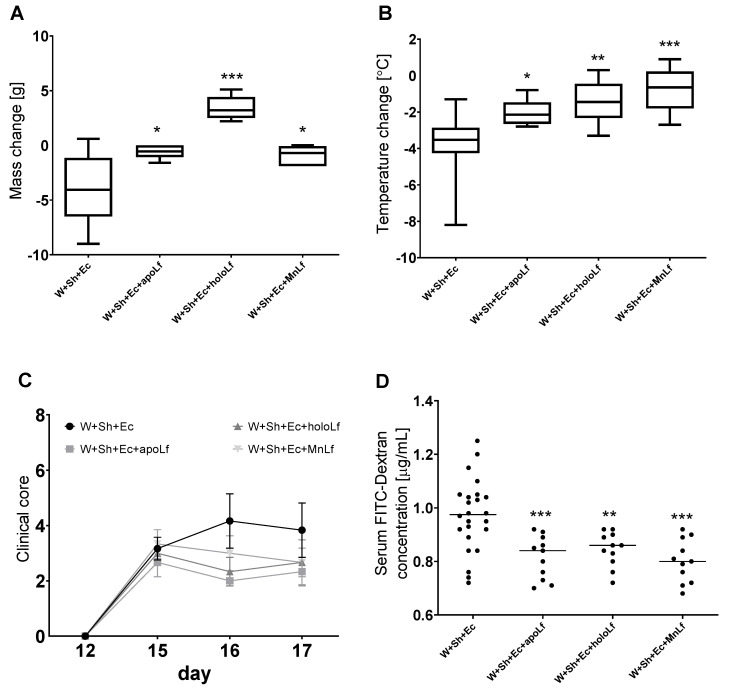
Clinical parameters of animals in tested groups with lactoferrin supplementation: change in weight (**A**) and temperature (**B**) between the day of necropsy (day 17) and weaning (day 12). (**C**) Clinical scores (reduced motor activity, lethargy, shivering, piloerection) were assessed on days 12, 15, 16 and 17. Dextran concentration was calculated using standard curve for FITC-Dextran (**D**). * *p* < 0.05, ** *p* < 0.01, *** *p* < 0.001 vs. W+Sh+Ec. W+Sh+Ec-target model group weaned with administration of both intraperitoneal *S. haemolyticus* 186 and oral *E. coli* 3A/1. W+Sh+Ec+apoLf-p.o. metal-depleted apoLf, W+Sh+Ec+holoLf-p.o. iron-saturated holoLf, W+Sh+Ec+MnLf-p.o. manganese-saturated MnLf.

**Figure 4:**
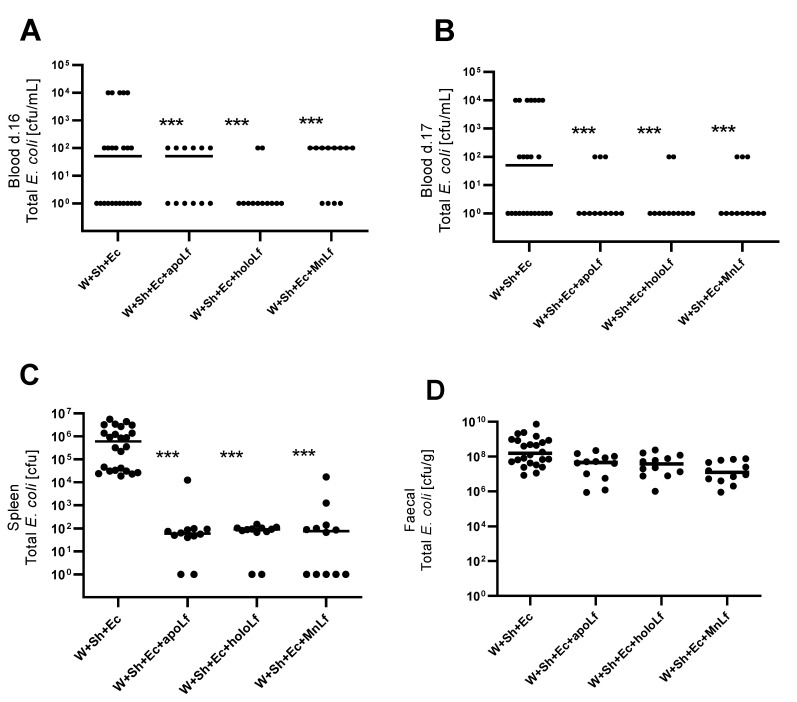
Population numbers of total E. coli for groups with lactoferrin supplementation in blood d. 16 (**A**), blood d. 17 (**B**), spleen (**C**) and feces (**D**). *** *p* < 0.01 vs. W+Sh+Ec group. W+Sh+Ec-target model group weaned with administration of both intraperitoneal *S. haemolyticus* 186 and oral *E. coli* 3A/1. W+Sh+Ec+apoLf-p.o. metal-depleted apoLf, W+Sh+Ec+holoLf-p.o. iron-saturated holoLf, W+Sh+Ec+MnLf-p.o. manganese-saturated MnLf.

**Figure 5:**
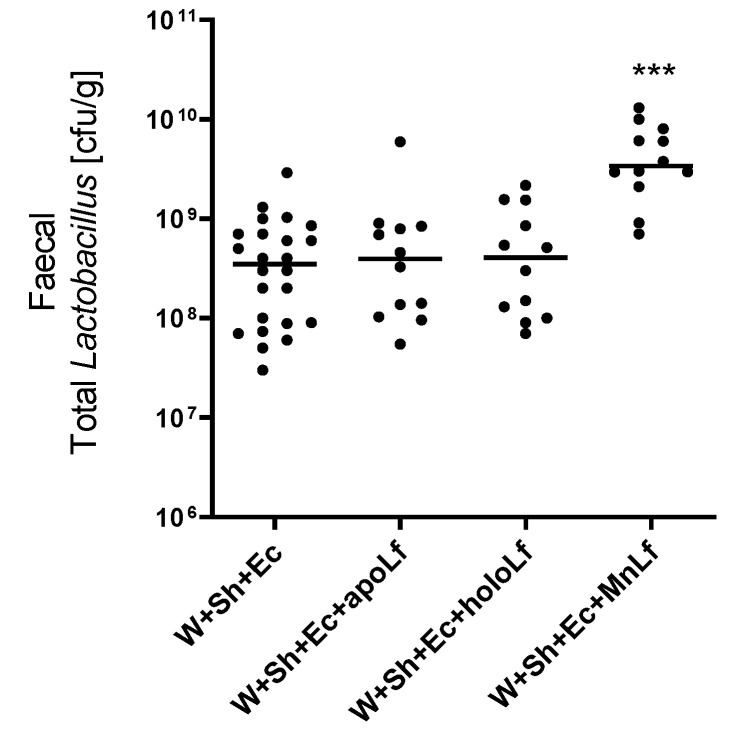
Fecal population numbers of total lactobacilli for groups with lactoferrin supplementation. *** *p* < 0.01 vs. W+Sh+Ec and W+Sh+Ec+apoLf and W+Sh+Ec+holoLf. W+Sh+Ec-target model group weaned with administration of both intraperitoneal *S. haemolyticus* 186 and oral *E. coli* 3A/1. W+Sh+Ec+apoLf-p.o. metal-depleted apoLf, W+Sh+Ec+holoLf-p.o. iron-saturated holoLf, W+Sh+Ec+MnLf-p.o. manganese-saturated MnLf.

Oral administration of lactoferrin (no matter which form) seemed to be beneficial for the overall physiology of the tested animals. Negative changes in both mass (Figure 3A) and temperature (Figure 3B) in the course of the experiment were ameliorated in the groups in which lactoferrin was administered. There was also a slight decrease in the clinical scores of the lactoferrin-supplemented groups compared to the model (W+Sh+Ec) group, but no statistically significant changes were observed (Figure 3C). Orally supplemented lactoferrin mitigated the bacterial translocation of *E.coli* from intestinal lumen to blood (Figure 4A,B) and spleen (Figure 5C). We observed a significant decrease in total *E. coli* numbers in blood and spleen in the case of all tested forms of lactoferrin (regardless of metal saturation). At the same time, fecal levels of *E. coli* remained the same (Figure 4D) while population numbers of lactobacilli were significantly increased in the group supplemented with manganese-saturated lactoferrin (Figure 5). This phenomenon could be attributed to the release of manganese from this form of lactoferrin and stimulation of growth of these probiotic bacteria. FITC-Dextran levels confirm the previous results—translocation of this marker was significantly reduced in lactoferrin-supplemented groups compared to the model group (W+Sh+Ec, Figure 3D).

## 4. Discussion

Herein, we present the development of a novel animal model of neonatal sepsis induced by bacterial translocation and the application of such a model to elucidate the effect of oral supplementation with various forms of lactoferrin. We identified that lactoferrin saturated with manganese significantly increases the *Lactobacillus* bacterial population, which contributes to the fortification of the intestinal barrier and inhibits the translocation phenomenon. The acquired knowledge can be used to limit the development of sepsis in newborns in hospital neonatal intensive care units.

Neonatal sepsis remains a significant cause of mortality and morbidity for infants worldwide. Rapid deaths of newborns primarily result from the extremely short time that can elapse from the onset of an acute infection to the development of sepsis [2]. Isolation of the preterm newborns from the mother is a colossal stress factor affecting temporary intestinal translocation. This physiological phenomenon is considered desirable from an immunological perspective, leading to the development of immune tolerance to specific components of gut microflora colonizing a newborn’s digestive tract [2,14]. However, the colonization of a newborn’s GI tract with some maternal etiological factors (e.g., *Streptococcus agalactiae*) might facilitate the development of early onset sepsis occurring up to few days after delivery. This form of infection appears to be strongly associated with maternal microflora and perinatal disorders. Therefore, proper perinatal antibiotic therapy significantly reduces this type of condition. Late-onset sepsis, i.e., an acute infection leading to death up to several days after delivery, is mainly associated with lengthy hospitalization and complex diagnostic procedures [3,4]. Hospital-derived coagulase-negative staphylococci with various resistance mechanisms are the most frequent etiological factors causing LOS [15,16]. Recently, however, epidemiologists have observed an alarming occurrence: LOS is often caused by aerobic Gram-negative rods, particularly *Escherichia coli*, which are more often responsible for the death of newborns in comparison to staphylococcal sepsis [4,17].

Developing a suitable animal model corresponding to the physiological changes observed in a newborn’s organism during LOS has allowed research to verify the possibility of regulating this phenomenon through targeted nutrition. Both the advancement and LOS course result from many factors affecting a newborn’s organism, such as infections caused by highly virulent microorganisms and certain organ malfunctions occurring after an extended hospitalization. There were several unique features required for the establishment of the LOS animal model. In our study, we adapted a model of rat pups that were weaned on day 12 post-birth (group: W), when they were able to eat on their own. It has been revealed that weaning-related stress contributes to intestinal leakages and, subsequently, to gut–spleen translocation of *E. coli*, evidenced by positive cultures of *E. coli* in crushed fragments of the spleen (2 cases per 6). Interestingly, despite translocation, no other deteriorative parameters affecting the animals’ health were observed.

The *E. coli* strains isolated from the organs had the same drug resistance pattern as well as DNA profile as an *E. coli* originating from pups’ feces collected during necropsy on day 17 (data not shown) [18]. This was in line with the observation by Moussaoui team that Wistar puppies exposed to limited nesting stress from 2 to 10 days after birth exhibit increased total intestinal permeability [8]. Other studies also investigated a change in the intestinal barrier at weaning in the maternal separation model [19]. However, the presence of gut bacteria in the blood is only temporary and is followed by a quick clearance [5]. The literature has shown that bacterial translocation in rats is a common early life phenomenon. It is most probably related to the activity of submucosal macrophages, which, without initiating a pro-inflammatory immune response, effectively kill bacteria that have managed to overcome the intestinal epithelial barrier [20]. Based on our further research, it has been observed that the condition of animals was altered in the remaining two groups: W+Sh and W+Ec, in which the animals were subject to two stress factors acting simultaneously. In the W+Sh group, weaning served as the first stress factor, and one dose of a pure culture of *Staphylococcus haemolyticus* was administered intraperitoneally as a second stress factor. In the W+Ec group, weaning served as the first stress factor; however, a pure culture of *E. coli* was administered orally as a different second stress factor.

Even though we observed a slightly higher number of positive blood cultures of intestinal *E. coli* in the W+Sh group on day 16, we did not notice a significant deterioration in the animals’ health. Similar results were obtained in the W+Ec group, indicating that newborn rats can deal with two simultaneous stress factors quite effectively with only a minor deterioration in clinical parameters.

A completely different situation was observed in the last study group, where rats were subject to synchronized three-stressor action (W+Sh +Ec). When three stress factors acted synergistically, the intestinal barrier broke down, and Gram-negative rods (a dominant component of the intestinal microbiota) entered the bloodstream, causing bacteremia and endotoxemia. It subsequently led to a significant deterioration in all live, clinical and microbiological parameters. Based on these studies, it seems that the population of coagulase-negative staphylococci, which cause systemic infection symptoms that weaken the immune system, assists the phenomenon of translocation only when the newborn’s intestine presents an increased population of Gram-negative bacteria. Thanks to this model, bacterial translocation and its consequences for a newborn can be analyzed in detail.

Our further endeavors focused on elucidating which factors may determine the growth of the *E. coli* population in the neonatal gut. According to the current literature, ferric ions constitute a significant factor promoting the development of most Gram-negative rods. These ferric ions may appear in the intestine as a result of a small blood vessel rupture occurring throughout a digestive tract of a newborn—this phenomenon frequently occurs in necrotizing enterocolitis (NEC) [21,22]. Furthermore, stimulating the epithelial cells to produce tight junction proteins and enhancing the intestinal barrier might counteract bacterial translocation [23].

Theoretically, lactoferrin (Lf)—a protein found in colostrum and breast milk—can actively chelate Fe ions and has antimicrobial, anti-inflammatory and immunoregulatory properties [5,24]. Lf is natural compound that has been shown to play a key role in counteracting inflammatory homeostasis disorders associated with both aseptic and septic pathological conditions [25].

Feeding of Lf to neonatal rats before inducing an intestinal infection with *E. coli* was shown to enhance survival of the rat pups, reduce infection of the jejunum and ileum [26] and limit *E. coli*-related translocation to the liver and blood [27]. Lactoferrin (especially apolactoferrin) can sequester free ferric ions in the intestine and is therefore bacteriostatic for Gram-negative rods [11]. Additionally, lactoferrin can bind LPS, and thus inhibit the immunostimulatory effect of endotoxin on immune cells [23]. Research concluded that lactoferrin supplementation reduced the risk of late-onset infection by 40% and necrotizing enterocolitis by 60% [28]. Other studies showed that enteral lactoferrin supplementation significantly reduced late-onset sepsis incidence in very low birth weight newborns (VLBW) [29]. Another study demonstrated that stress factors such as a maternal separation (for 3 h per day from days 4 to 19 of life) of neonatal rats disrupted normal bacterial colonization of the gut, impaired colonic physiology and reduced host defenses. Adherent bacteria were dramatically increased in the colon of separated rats; in contrast, the number of lactobacilli was significantly reduced. Treatment with *Lactobacillus* strains restored normal gut function [19].

Therefore, it seems that the newborn’s organism, when exposed to at least three stress factors concurrently, cannot react appropriately, which leads to a rapid loss of intestinal barrier integrity and the development of LOS. Finding natural substances that will seal the newborn’s intestine should be a priority because any extended stay in the hospital will always lead to the phenomenon of translocation. It seems that lactoferrin containing Mn ions may be such a substance.

It is crucial to understand the limitations of the established model and performed experiments. The first stress factor—weaning—actually has two important components: weaned pups are deprived of milk (cease of breastfeeding) as well as the physical inter-action with their mother (cease of bonding). Further studies should be conducted to elucidate the impact of these two ‘subfactors’ by, e.g., oral supplementation of the weaned pups with animal milk/formula. What is more, due to the constraints of our study, we have focused on differentiating between the most ‘extreme’ species of lactoferrin—one devoid of metal ions (apolactoferrin), one loaded with iron (hololactoferrin) and finally the manganese-saturated one (MnLf). Naturally, it would be interesting to verify what effect the natural (10–20% saturated) lactoferrin would exert on the established model. Lastly, in this study, we focused on two groups of bacteria relevant for neonatal sepsis (*Enterobacteriaceae* and lactobacilli), and we were interested in their colony-forming ability. Our future studies will also include molecular analysis of fecal DNA using next-generation sequencing methods for more complex results.

The developed model allows us to analyze the translocation process taking place in the organism of newborns under the influence of several factors at the same time, all of which are relevant for the physiology and pathology of the neonatal gut. Separation from the mother, possibility of nosocomial infection and colonization of the intestine with potentially pathogenic bacterial species are all risk factors for the neonatal sepsis originating from the gut. Furthermore, our model allows the search for active substances that, when administered orally, have potent properties to counteract bacterial translocation by restoring the integrity of the neonatal intestinal barrier.

## Figures and Tables

**Table 1:** Procedures performed on animals in individual study groups during the experiments.

Day	Procedure	NW	W	W+Sh	W+Ec	W+Sh+Ec	W+Sh+Ec	W+Sh+Ec	W+Sh+Ec
							+apoLf	+holoLf	+MnLf
12	Weaning	−	+	+	+	+	+	+	+
	M+T	+	+	+	+	+	+	+	+
14	Lactoferrin	−	−	−	−	−	+	+	+
15	*S. haem.* 186 *i.p.*	−	−	+	−	+	+	+	+
	M+T	+	+	+	+	+	+	+	+
	Blood collection	+	+	+	+	+	+	+	+
	Lactoferrin	−	−	−	−	−	+	+	+
16	*E. coli* 3A/1 *p.o.*	−	−	−	+	+	+	+	+
	M+T	+	+	+	+	+	+	+	+
	Blood collection	+	+	+	+	+	+	+	+
	Lactoferrin	−	−	−	−	−	+	+	+
17	M+T	+	+	+	+	+	+	+	+
	FITC-Dextran	+	+	+	+	+	+	+	+
	Necropsy	+	+	+	+	+	+	+	+

[+] performing an action, [−] no action. M+T-body mass and temperature measurement, NW-non-weaned control group, W-weaned group, W+Sh-weaned pups with intraperitoneal administration of *Staphylococcus haemolyticus* 186, W+Ec-weaned pups with oral administration of *Escherichia coli* 3A/1, W+Sh+Ec-target model group weaned with administration of both intraperitoneal *S. haemolyticus* 186 and oral *E. coli* 3A/1. W+Sh+Ec+apoLf-p.o. metal-depleted apoLf, W+Sh+Ec+holoLf-p.o. iron-saturated holoLf, W+Sh+Ec+MnLf-p.o. manganese-saturated MnLf.

## Data Availability

The data presented in this study are available on request from the corresponding author.
